# Gap analysis and implications for seasonal management on a local scale

**DOI:** 10.7717/peerj.5622

**Published:** 2018-09-20

**Authors:** Li Yang, Baofeng Zhang, Xinrui Wang, Yueheng Ren, Jinlin Chen, Chao Zhang, Yongpeng Xia, Yuankun Li, Jianguo Sun, Jiangang Guo, Weijia Wang, XiaoFeng Luan

**Affiliations:** 1 School of Nature Conservation, Beijing Forestry University, Beijing, China; 2 Zoological Society of London, IUCN SSC Pangolin Specialist Group London, London, UK; 3 School of Life Science, Sun Yat-sen University, Guangzhou, China; 4 Chinese Research Academy of Environmental Sciences, Beijing, China; 5 School of Life Sciences, Beijing Peking University, Beijing, China; 6 Hebei Wulingshan National Nature Reserve, Chengde, China

**Keywords:** Protected area, Local scale, ENM

## Abstract

**Background:**

Identifying biodiversity hotspots on a local scale, using multiple data sources, and ecological niche modeling, has the potential to contribute to more effective nature reserve management.

**Methods:**

In this study, we used infrared-triggered camera trapping, field surveys, and interviews to create a dataset on the distribution of species (mammals and birds) in Hebei Wulingshan Nature Reserve (Hebei Province, China).

**Results:**

We identified 101 species (14 orders, 38 families), 64 of which (2,142 effective records) were selected for environmental niche modeling. All results were reclassified into three groups: “priority areas” (areas including the potential distributions of over 80% of species), “important areas” (those with 50% of species), and “normal areas” (all other areas). Our results show that priority areas (1.31–1.82 km^2^) and important areas (7.73–21.44 km^2^) for conservation were mainly covered by the core and experimental zones of the reserve; additionally, a kilometer-wide margin around the outside of the nature reserve seems to be important to maintaining biodiversity.

**Discussion:**

We close by suggesting some actions for enhancing conservation of biodiversity in the reserve, including monitoring, strengthen law enforcements, introducing popular science, and co-operating with local people.

## Introduction

Protected areas, particularly nature reserves, are a basic unit for maintaining the world’s biodiversity. Numerous studies have focused on biodiversity conservation on a regional or even global scale, while relatively few have investigated this issue on a local scale (Chen, Luo & Liu, 2017; Li et al., 2017; Luo, Jiang & Tang, 2015; Pacifici et al., 2015; Scherrer et al., 2017). In fact, research on biodiversity issues on a local scale may provide more useful information to the managers of nature reserves, like seasonal management. There is thus a gap between the biodiversity research currently being undertaken and the needs of those who apply the results of this research to the management of protected areas, especially in Southeast Asia and China.

Identifying conservation gap on a local scale can be helpful to management, which need research efforts. Biodiversity research on a local scale is generally hampered by insufficient data (e.g., a lack of effective records, camera sites, high-resolution environmental variables, and/or funding). In response to these problem and resources limitations for conservation, research efforts need to focus on with data shortage. Wildlife monitoring data from infrared-triggered camera trapping, field surveys, and interviews have been successfully applied in numerous conservation studies (He et al., 2016; Lescureux et al., 2011; Li et al., 2010; Newton et al., 2008; Rovero & Marshall, 2009; Villette et al., 2016). Combining data from multiple sources into a single dataset can ameliorate the data shortage that often limits local studies. In addition, primary data on a local scale is often incomplete and spatially biased, a problem that can be mitigated through the use of ecological niche modeling (Hammitt, Cole & Monz (2015); Hortal et al., 2008; Marks (2012); Yang et al., 2016; Zhang et al., 2016). Such modeling has proven to be an effective tool for reducing the data limitations of traditional methods for measuring biodiversity (Carretero & Sillero, 2016; Elith & Leathwick, 2009; Ranjitkar et al., 2016; Thuiller et al., 2009). Combining multiple data sources on a local scale with ecological niche modeling offers a potential solution to such limitations.

Previous studies show that invertebrates in general are appropriate indicators of ecosystem integrity, because they are more strongly associated with environmental factors than biological factors, like predation and parasitism (Carignan & Villard, 2002). Ecosystem integrity is one of the most important goals for environmental management on protected areas. However, conservation biology requires more specific information than a list of species to meet the increasing need in ecological management (e.g., seasonal management). To achieve this goal, we focused on mammal’s and bird’s biodiversity, and tried to obtain effective information for a protected area in a local scale. Therefore, we (1) collected and combined data from multiple sources and created species occurrence datasets; (2) constructed climate data to represent environmental variables at high-resolution; (3) estimated potential species distributions by ecological niche modeling (ENM, by BIOMOD2) and identified possible hotspots to support environmental management for a protected area on a local scale.

## Material and Methods

### Study area

**Figure 1:**
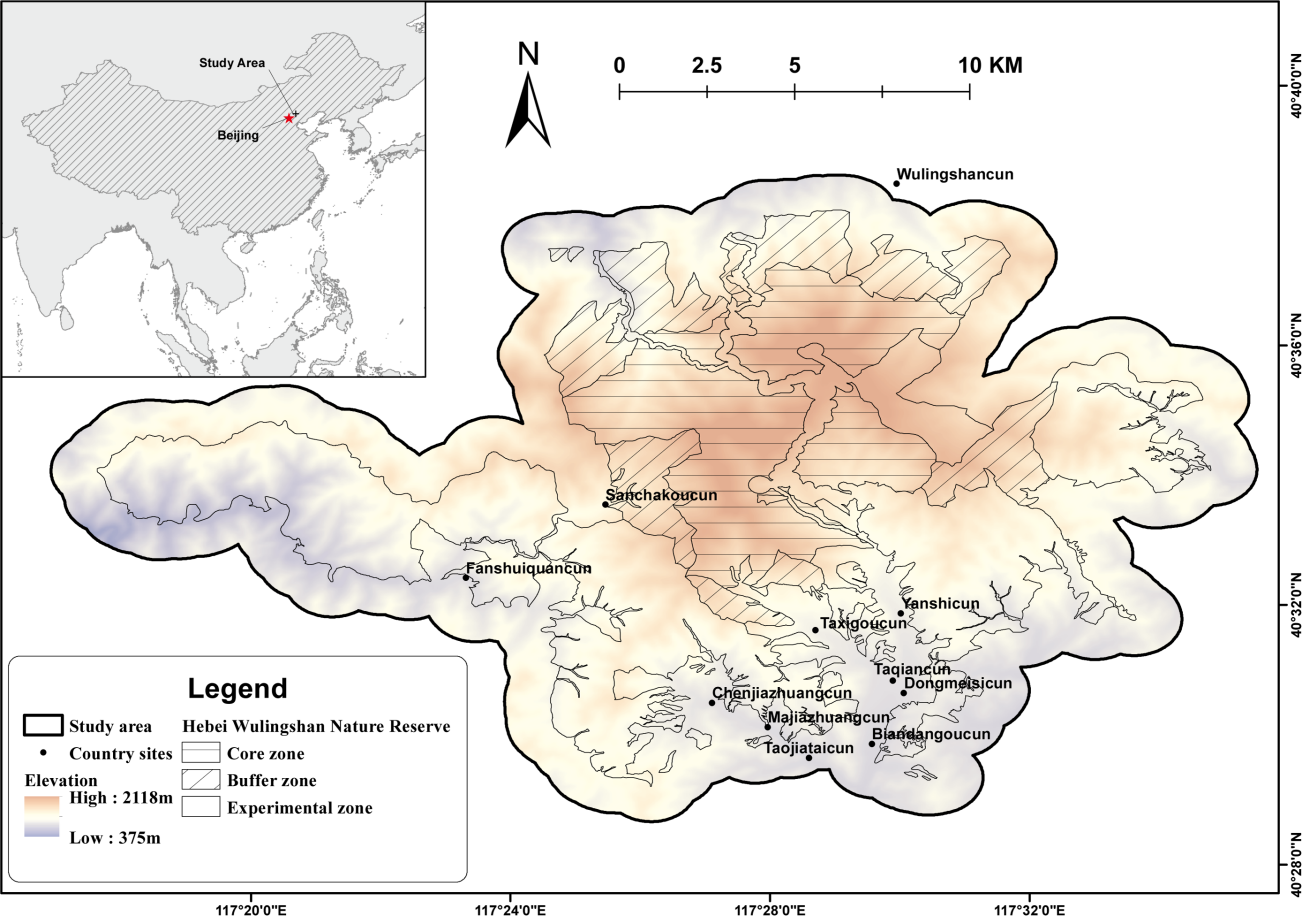
Topographic map of Hebei Wulingshan National Nature Reserve showing the study area.

The study area is located in Hebei Wulingshan Nature Reserve in Yanshan Mountains (117°17′E–117°35′E, 40°29′N–40°38′N, Hebei Province, China; Fig. 1). The reserve covers an area of 142.47 km^2^, of which the Core Zone is 37.95 km^2^, the Buffer Zone is 24.04 km^2^, and the Experimental Zone is 80.48 km^2^. Elevation ranges varies from approximately 375 to 2,118 m. The average annual temperature is 7.6 °C (3.8–11.4 °C), with the lowest temperatures reaching −15.6 °C in January and the highest temperatures reaching 17.6 °C in July. The average annual rainfall is about 763 mm (436–1,101 mm). The habitat changes along with the elevation: temperate broadleaf and subalpine coniferous forests are mainly present at low and mid-elevation; and subalpine scrub meadows at high elevation.

### Data collection

Our data were collected from September 2015 to June 2018 (with a research permission approved by Hebei Wulingshan National Nature Reserve) using infrared-triggered camera trapping, field surveys, interviews, and specimen records (historical records) in the protected area (see Appendix I for details). Conflicting records (*n* = 1,468) with unsubstantiated metadata, such as those lacking relevant or detailed descriptions, were excluded from the analysis, and effective records (*n* = 2,142) were selected for modeling. We divided animals into three groups: resident (include mammals and resident birds), summer (summer residents), and winter (winter residents).

### Camera records (mainly focus on mammals)

According to previous research, Hebei Wulingshan Nature Reserve was divided into one km^2^ blocks by ArcMap 10.2 (totally 142 blocks; cell size 1 × 1 km). Some blocks were excluded because of topography limitation for research. Only blocks in Core Zone and Buffer Zone were selected for research. Each block had one camera unit for at least 4 months. Finally, 56 sites were obtained (Fig. S1 in Appendix I).

Camera units (SG-990V, 30; Digicool SG-680V, 20; Digicool SG-009, 20; Digicool, Beijing, China) were attached to trees 80–120 cm above the ground, and three to five m from a trail or point where animal movement might be expected. Moreover, the distance between each camera was far more than 400 m (mean: 558 m, range from 354 to 766 m). For each site, GPS location, elevation, slope, aspect, and habitat (e.g., vegetation cover, forest type, and human influence) were recorded. Three photos (1,200 PX) and a 15 s film (1,080 P) were taken for every trigger. Camera units were also set with a 2 min delay between each photograph and set for 24 h monitoring. At the end of each monitoring session, the units were tested to confirm that they were still operational and had unexposed frames; otherwise, the date on the last photograph was taken as the last operational date.

A monitoring network of infrared cameras has been created for Hebei Wulingshan Nature Reserve from September 2015 to December 2015; and from March 2016 to June 2018. A sampling effort of 22,379 camera-days across 56 sample sites was achieved (Fig. S1 in Appendix I). A total of 1,736 records for 15 species were obtained. We defined detection at a sample point as one individual photographed during a 30 min period. If more than one individual were identified on a single photograph, we considered this one detection. All detections were summed for each camera site (Jia et al., 2014; Li et al., 2010, 2014; Liu et al., 2013; Zhang et al., 2012). Then, species were identified using several books (Jiang et al., 2015; MacKinnon, Phillipps & He, 2000; Smith & Xie, 2009; Zheng, 2011) and databases (Animal Diversity Web, http://animaldiversity.org/).

Finally, 548 effective records for 15 species (two birds; 13 mammals) were selected.

### Bird survey

Bird survey was launched at four periods (April 2016; July 2016; October 2016; January 2017) due to time and access constraints. A total of 14 transects were designed for bird surveys covering important habitats (e.g., forestry, mesophorbium, farmland) and elevation range in Hebei Wulingshan Nature Reserve (Table S2; Fig. S2 in Appendix I). Length of transects ranged from 2.5 to 12.7 km.

Four man a day surveyed each transect starting survey at 8 am and finishing end at 18 pm. Data on bird species present were collected by direct observation and records of calling birds. Bird names and species-level taxonomy generally followed several books (MacKinnon, Phillipps & He, 2000; Zheng, 2011) and databases (Birdlife International, Cambridge, UK, http://www.birdlife.org/). IUCN Red List categories followed International Union for Conservation of Nature (IUCN, http://www.iucnredlist.org) CITES categories were obtained from Species+ (https://www.speciesplus.net/); red list categories were obtained from Red list of China’s Biodiversity (http://www.zhb.gov.cn/gkml/hbb/bgg/201505/t20150525_302233.htm). For each species, we considered one only effective record if the distance between two records was less than 30 m.

Finally, 1,449 effective records for 60 species (27 resident birds, 18 summer birds, and five winter birds) were selected.

### Interview

Knowledge from local people has been proved to be an effective tool for animal survey. An interview was carried out between November 2016 and January 2017, covering our study area (Fig. S3 in Appendix I). We conducted semi-structured interviews without precise, pre-determined questions so that interesting lines of discussion could be pursued. Open questioning was employed wherever possible, to avoid leading the interviewee into only one answer. All the questions addressed on the distribution, historical range, and last records for animals in Hebei Wulingshan Nature Reserve. We used the photographies for identification. Each dialog was recorded by one of the interviewers and uncertainties were clarified immediately after the interview. Only records with detail location information (including the name of a specific place in Hebei Wulingshan Nature Reserve) were selected. Then, these records were crosschecked with records from camera sites and bird surveys. Some records were excluded if they were not recorded by our field investigation. Finally, 288 local people from 11 villages in our study area were interviewed. In order to reduce bias, interview records which were confirmed by the camera trap or bird survey were selected. A total of 112 records (including mammals and birds) were selected for research.

In addition, we also collected specimen records from Hebei Wulingshan Nature Reserve (These museum specimens’ data was provided by Hebei Wulingshan Nature Reserve). A total of 33 records (13 mammals and 20 birds) were selected for research.

### Environmental data

Climate data were downloaded from Climate AP v2.03 (http://climateap.net/; Wang et al., 2012); see Appendix I for details). Environmental variables were calculated by interpolation of complex multivariate data using thin-plate smoothing splines with ANUSPLIN version 4.36. Based on previous research (Feng et al., 2015; Petitpierre et al., 2016) and survey results, nine environmental variables (two topography variables and seven climate variables) which can present species physiological limits were selected. We divided environmental variables into three groups: annual, summer, and winter (see details in Appendix I).

### Modeling

Previous researches have been show that sometimes, sampling effort is associated with the proximity to roads (cites) (Beck et al., 2014; Marks, 2012; Ponder et al., 2001). Therefore, we obtained information about roads close to the Nature Reserve. Then we created 2,000 random points within a buffer area of two km wide around the considered road (only main roads that have existed for more than 30 years were selected).

Collinearity problems can lead to bias. Given the problems in predicting, it is necessary to assess collinearity both in training and in prediction data sets. Following the suggestions described in Dormann et al. (2013), we used a Variance Inflation Factor (VIF) analysis in this research. This analysis was calculated in R and the threshold was set as 10 (only VIF values lower than 10 were selected). For each species, different variables were selected for modeling (Ranjitkar et al., 2016).

We estimated the potential distribution for species using BIOMOD2 implemented in the BIOMOD2 R package (version 3.3-7) (Phillips, Anderson & Schapire, 2006; Thuiller et al., 2009) and used five modeling techniques: generalized additive modeling (Hastie & Tibshirani, 1986), generalized linear modeling (GLM) (Thompson et al., 1985), multivariate adaptive regression splines (MARS) (Friedman, 1991), random forest (RF) (Breiman, 2001), and maximum entropy (MAXENT, Phillips) (Phillips, Anderson & Schapire, 2006). All techniques were applied to presence data for each species and to the pseudo-absences for the models developed in BIOMOD2 (use default option). Model parameters (GLM, MARS, and RF) were tuned by BIOMOD2, except MAXENT, Phillips which was tuned by ENMeval package (Aiello-Lammens et al., 2015; Radosavljevic, Anderson & Araújo, 2014). For each species, each ENM was evaluated measuring the true skill statistic (TSS) (Allouche, Tsoar & Kadmon, 2006), with 10 evaluation runs. For the TSS, 50 modeling evaluation results were obtained, and the average of the TSS was set as the threshold for building the ensemble models. Then, the potential distribution was calculated from an ensemble set of models or predictions. To transform the models from environmental suitability into presence–absence distributions, we used the threshold (*P*, cut-off) calculated by BIOMOD2. From this, all outputs were divided into two groups; outputs above the threshold (>*P*) were grouped as “present,” while all other values (<*P*) were considered “absent.” Finally, the potential distribution through time were obtained.

### Conservation

We investigated species distributions in four scenarios: all species, resident (mammals and resident birds), summer (mammals and summer birds), and winter (mammals and winter birds). We identified local biodiversity hotspots according to the degree of overlap of potential distributions. Then each layer was reclassified into three groups: “priority areas” (areas including the potential distributions of over 80% of species), “important areas” (those with 50% of species), and “normal areas” (all other areas). Then we identified conservation gaps on a local scale for each scenario. Spatial analyses were conducted in ArcGIS version 10.2 (ESRI Inc., Redlands, CA, USA) and SPSS for Windows version 20.0 (SPSS Inc., Chicago, IL, USA).

## Results

### Data collection and sample effective

We identified 101 species from 14 orders and 38 families in Hebei Wulingshan Nature Reserve, including 13 mammals (from nine families in five orders) and 88 birds (resident: four orders, 12 families, 27 species; summer: four orders, nine families, 19 species; winter: two orders, four families, five species; other (stragglers and travelers): four orders, 13 families, 37 species). Of these species, two were listed as vulnerable (VU) in the IUCN red list, and three in CITES (Appendix II); eight were listed in China’s biodiversity red list (two VU; six NT). To reduce bias, we excluded stragglers and travelers from our analysis (see Appendix II for details). Finally, 64 species were selected for modeling.

**Figure 2:**
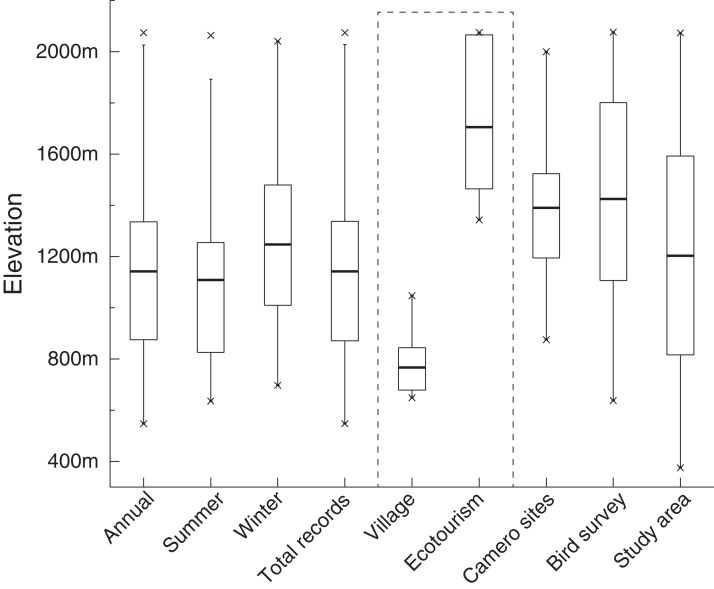
Samples effected in our research. Dash line highlights human influence in this study. On the left side of dash line, the elevation range for occurrence points for three groups were presented, while elevation range for our survey and study area were shown in the right side.

Our results show that elevation ranges of occurrence points for each species were similar to the elevation range of the study area, especially from 800 to 1,500 m. Our research (including camera records and interviews in villages) covered almost the entire elevation range of the study area. Human influence, including villages and ecotourism, mainly ranged from 700 to 850 m, and from 1,500 to 2,100 m, respectively. Occurrence points mainly covered a mid-elevation range, perhaps avoiding human influence (Fig. 2).

### Model results

We created 64 pseudo-absence datasets for individual species. The number of records per species ranged from 10 (*Cuculus saturatus*) to 113 (*Parus songarus*), with an average of 33.5. On average, most internal evaluations provided fair or good results, with most TSS values being >0.40 (see Appendix II for details). The threshold ranged from 0.57 to 0.84. All fair and good results were selected for building the ensemble models. Finally, TSS for each species ranged from 0.68 to 0.94, providing good results.

### Potential distribution

**Figure 3:**
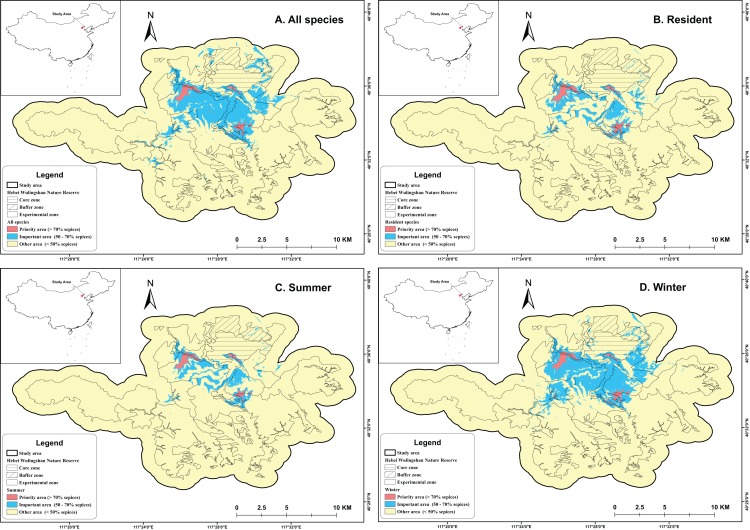
Study area with hotspots identified by four different scenarios. (A) presents hotspot of all species in the study area; (B) presents hotspot of resident species in the study area; (C) presents hotspot of summer species in the study area; (D) presents hotspots of winter species in the study area.

The potential distributions of individual species ranged from 7.64 to 63.42 km^2^ (24.79 km^2^ on average). The mean distribution of year-round residents, including both mammals and birds, was 26.80 km^2^; that of summer species was 20.71 km^2^, and that of winter species was 24.34 km^2^. The average distribution of all mammals was 45.40 km^2^, while that of birds was 20.04 km^2^ (Fig. 3). The *Garrulax davidi* had the largest potential distribution, while the *Streptopelia chinensis* had the smallest. Elevation at which each species (including mammals and birds) was found ranged from 812 to 1,608 m. The *Prunella collaris* was found at the highest elevation, while the *Streptopelia chinensis* was found at the lowest. Mammals were found at elevations between 1,224.35 and 1,471.94 m (1,382.92 m on average), while the ranges of birds were 812–1,515.6 m (1,144.8 m), 913.2–1,608 m (1,213.16 m), and 1,278.34–1,425.51 m (1,371.59 m), for resident, summer, and winter species, respectively (see Table S1 in Appendix II for details).

**Figure 4:**
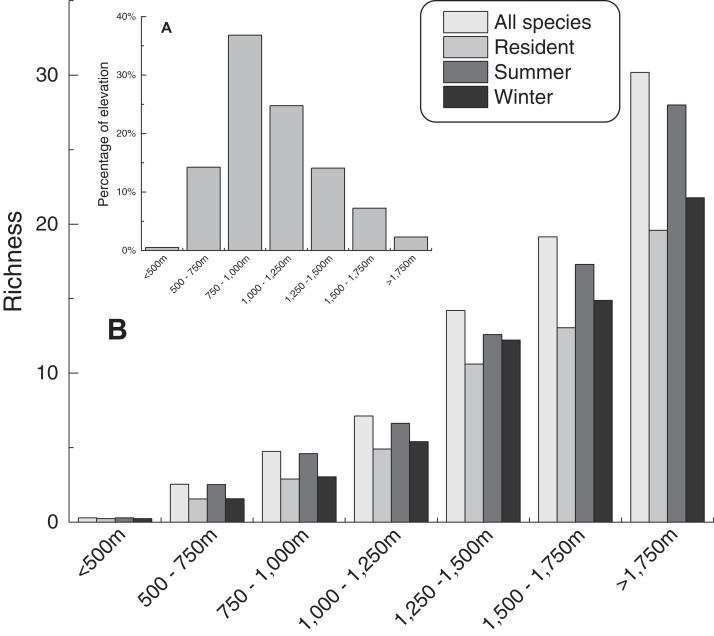
Species richness along with elevation. (A) presents the percentage of area on different elevation ranges; (B) presents species richness along with elevation on different groups.

The species richness of each group increases with increasing elevation (Fig. 4). The relationship between the area of different elevation categories and the richness of species is weak (Pearson test, values range from −0.38 to −0.40). This trend means that environmental management should focus on the area in higher elevation.

### Conservation

**Figure 5:**
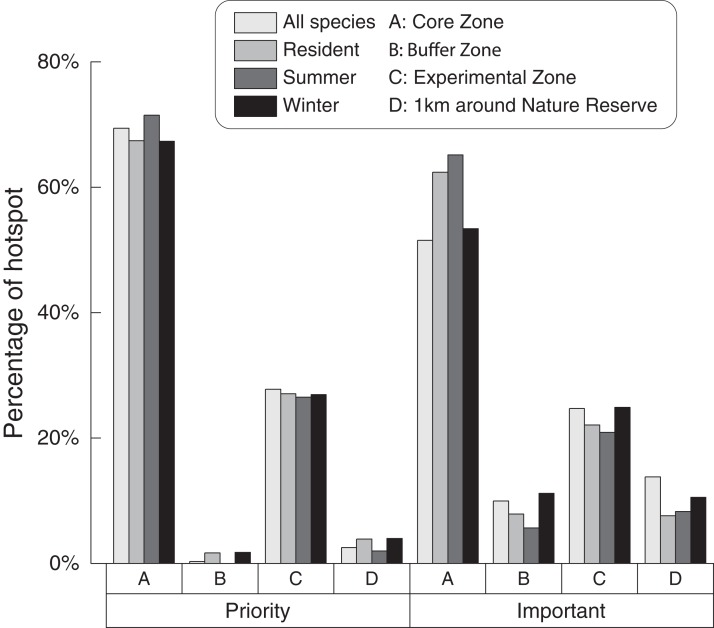
Relationship between nature reserve and hotspots under four scenarios.

The priority areas of the four scenarios ranged from 1.31 to 1.82 km^2^, while the important areas ranged from 7.73 to 21.44 km^2^. The nature reserve covered 96.92% of the priority areas and 89.93% of important areas in all scenarios. The core zone of the nature reserve covered about 68.91% of the priority areas and 58.12% of the important areas in all scenarios, while the experimental zone covered about 27.06% of the priority areas and 23.15% of the important areas. Our results also highlight that one km around the outside of the nature reserve’s perimeter seems to be important for maintaining biodiversity (priority area range from 1.97% to 3.97%, 3.08% on average; important area range from 7.62% to 13.82%, 10.07% on average) (Fig. 5; see Appendix II for details).

## Discussion

Results generated by combining data from multiple sources with species distribution modeling to estimate hotspots on a local scale are still restricted by practical and conceptual barriers, particularly by the shortage of useable data. Our results provide evidence that combining multiple data sources can offer an opportunity to identify hotspots (conservation gap) on a local scale and to fill the gap between the results of research and the needs of practitioners.

### Limitations

There are several limitations in this study that should be clarified: (1) the relationship between frequency of occurrence (photograph rate at a given site) and animal density remains unclear (Carbone et al., 2001; Li et al., 2010, 2014; Liu et al., 2013); (2) the effectiveness of different survey methods can vary depending on species, particularly for small mammals and birds; (3) variation in microclimate on different time frames may cause spatial changes in potential distribution (e.g., we only created variables for annual or seasonal population changes) (Broennimann et al., 2012; Sedgeley, 2001); (4) obtaining high-resolution environmental variables may create potential bias (Wang et al., 2012); (5) modeling limitations (e.g., part of a species’ fundamental niche may not correspond to any combination of environmental variables; species interactions may cause bias) (Cruz-Cárdenas et al., 2014; Elith & Leathwick, 2009; Guo et al., 2015; Maiorano et al., 2013); (6) human influence may be important but is difficult to quantify on a local scale.

However, occurrence records from different resource already contain potential information on human influences and habitat information, which could reduce bias caused by variables representative of the model. In addition, conservation action mainly addresses on species richness which can also reduce risk from modeling performance and potential bias for single species.

### Conservation implications

Our results show that mammals in Hebei Wulingshan Nature Reserve tend to live at middle to higher elevation (about 1,200–1,600 m), while birds cover a larger range of elevation. Species richness also presents a pessimism situation: higher elevation area tends to be more important while ecotourism presented in higher elevation will increase human influence. This difference may be associated with varying habitat needs and sensitivity to human influence: (1) human influence from villages may force animal to move into higher elevations and lead to lower records and richness in lower elevation. (2) Ecotourism in high elevation from June to October every year may affect the potential distribution in study area in two ways. One way may be negative effects that reduce the species occurrence, especially for mammals or some sensitive birds. Another way may be the optimistic influence that humans may change habitat patches, reduce predator (most mammals present in mid-elevation) or provide food (Hammitt, Cole & Monz, 2015). In fact, we tend to suggest that ecotourism provides a negative effect. Closing ecotourism in winter can reduce human influence significantly in higher elevation. Also, the animal potential distribution may be hampered by climate factors like low temperature, less food, and deep snow in winter in higher elevation. However, the mean elevation of occurrence points was higher in winter than in summer, which may imply that animals tend to go to the area with lower human influence and more food supply. Compared with priority and important conservation area, potential distribution in winter was larger than in summer.

Biodiversity hotspots are mainly located within the nature reserve, particularly in the core zone, which is under strict management. Species richness increasing along with elevation also needs more attention. However, high elevation area and biodiversity hotspots around the perimeter of the nature reserve need more attention.

**Figure 6:**
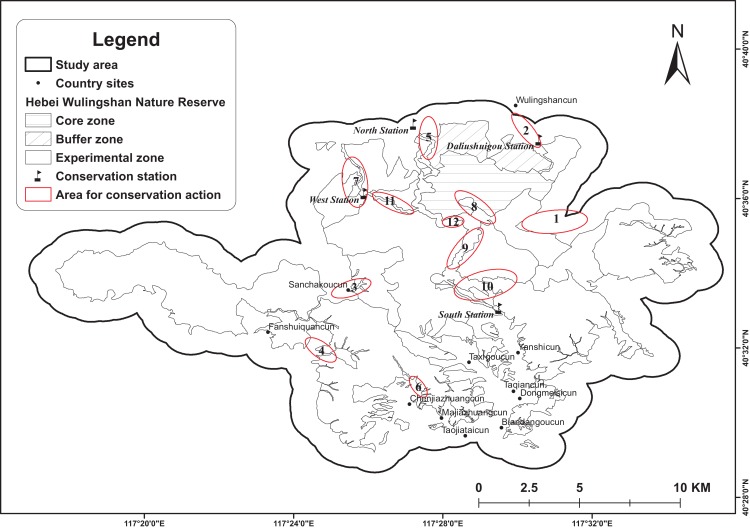
Conservation actions for national nature reserve. Six areas for conservation action addressed in the nature reserve, with another six areas around the nature reserve. Detailed suggestions for conservation action can be found in Appendix II.

We have therefore identified 12 areas that should be a priority for future management (Fig. 6) and suggest some immediate actions:

- **Monitoring:** (1) Area 1: Establish a monitoring network with camera traps, because of the proximity of this area to a forestry farm; (2) Areas 9–12: Patrol and monitor network (including camera sites in this research) can be considerable contribute to management, because management for human influence, or any conservation action would need support from this monitor system. (3) Areas 2, 4, and 6: Co-operate with local people (two villages near these area) to monitor species richness changes in these areas (Fig. 6).
- **Law enforcement:** Areas 7–12 should be priority area in summer, because effective enforcement will reduce ecotourism negative impact.
- **Popular science:** (1) Areas 2–4, 6, 7: Popular science should address the relationship between agriculture and conservation and focus on local people. (2) Other areas: Establish natural education system, mainly focused on conservation.
- **Co-operate with local people:** Promote ecological tourism and conservation-related enterprises that increase income for local people. This can change the life-style of local people and reduce disturbance of animal habitat around mountain areas. In addition, community co-management can be important to enhance conservation.

However, managers should share these monitor data with scientists. Then, they should try to answer some questions, like (a) why richness is increasing in winter, and what is the impact in Areas 1 and 2; (b) Areas 4 and 6 were important area for all species. Do these areas (4 and 6) need seasonal enforcement or specific conservation actions? In addition, management should encourage research which addresses the relationship between humans and animals in this national nature reserve, especially in high elevation areas.

## Conclusion

Research on a local scale may become an important part of conservation science in the near future, allowing conservation actions to focus on specific areas or time periods. Data gathered from such research will become a functional guide to conservation practitioners or management rather than mere theoretical support. An additional area for further research suggested by this study is the investigation of the relationship between nature reserves and the areas immediately surrounding them.

## Supplemental Information

10.7717/peerj.5622/supp-1 Supplemental Information 1 Appendix I. Method.

10.7717/peerj.5622/supp-2 Supplemental Information 2 Appendix II. Result.

10.7717/peerj.5622/supp-3 Supplemental Information 3 Raw data.

## Additional Information and Declarations

### Data Availability

The following information was supplied regarding data availability: The raw data are provided in a Supplemental File.
