# Supplementary material for: Gap analysis and implications for seasonal management on a local scale
Source: PeerJ. 2018 Sep 20;6:e5622. doi: 10.7717/peerj.5622 (PMC6151258; doi:10.7717/peerj.5622)
Supplement: Supplemental Information 1 [file peerj-06-5622-s001.docx]

**Appendix I method**


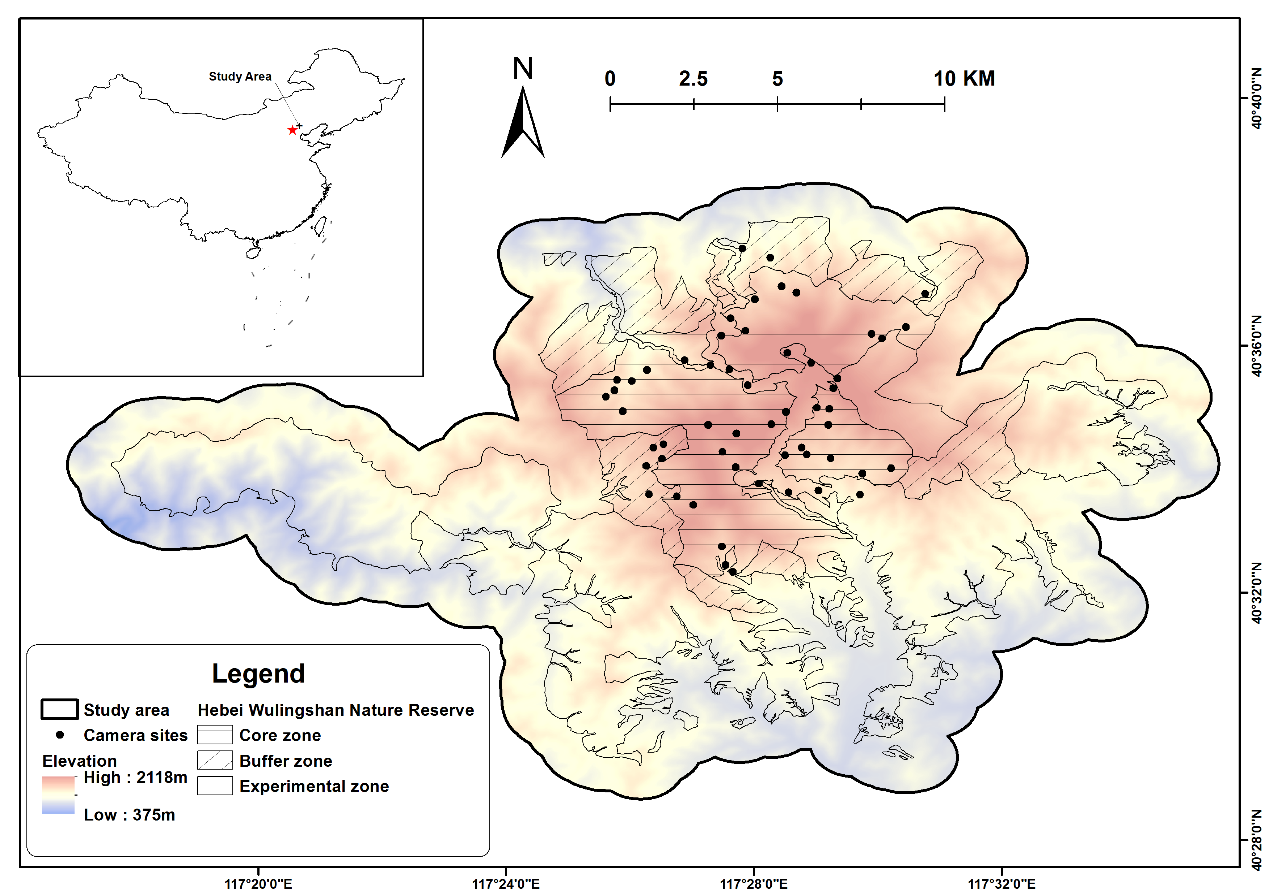


Figure S1 Camera sites in Hebei Wulingshan Nature Reserve


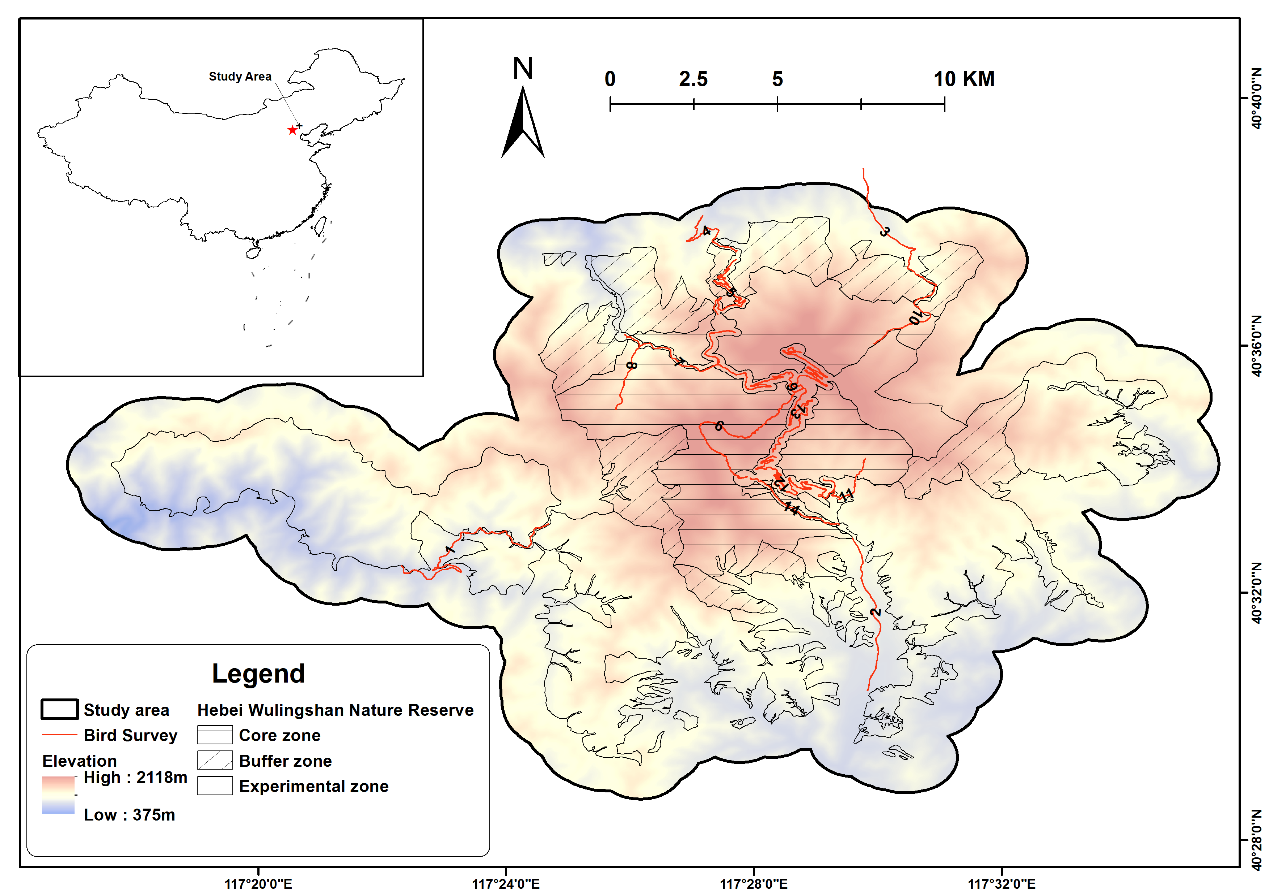


Figure S2 Transects for bird survey in Hebei Wulingshan Nature Reserve

TableS1 Detail information for camera sites in Hebei Wulingshan Nature Reserve

| ID | X | Y | ID | X | Y |
| --- | --- | --- | --- | --- | --- |
| 1 | 117.4836 | 40.5832 | 29 | 117.4503 | 40.5570 |
| 2 | 117.4869 | 40.5829 | 30 | 117.4299 | 40.5908 |
| 3 | 117.4866 | 40.5786 | 31 | 117.4378 | 40.5935 |
| 4 | 117.4873 | 40.5696 | 32 | 117.4756 | 40.5980 |
| 5 | 117.4795 | 40.5725 | 33 | 117.4820 | 40.5954 |
| 6 | 117.4809 | 40.5707 | 34 | 117.4890 | 40.5913 |
| 7 | 117.4957 | 40.5655 | 35 | 117.4880 | 40.5885 |
| 8 | 117.5035 | 40.5669 | 36 | 117.4750 | 40.5705 |
| 9 | 117.4951 | 40.5599 | 37 | 117.4840 | 40.5609 |
| 10 | 117.5011 | 40.6019 | 38 | 117.4610 | 40.5390 |
| 11 | 117.4983 | 40.6031 | 39 | 117.4580 | 40.5459 |
| 12 | 117.5075 | 40.6050 | 40 | 117.4590 | 40.5409 |
| 13 | 117.4752 | 40.5822 | 41 | 117.4650 | 40.5894 |
| 14 | 117.4396 | 40.5725 | 42 | 117.4600 | 40.5937 |
| 15 | 117.4423 | 40.5734 | 43 | 117.4550 | 40.5947 |
| 16 | 117.4543 | 40.5787 | 44 | 117.4480 | 40.5961 |
| 17 | 117.4619 | 40.5763 | 45 | 117.4710 | 40.6237 |
| 18 | 117.4713 | 40.5789 | 46 | 117.4740 | 40.6161 |
| 19 | 117.4419 | 40.5695 | 47 | 117.4780 | 40.6143 |
| 20 | 117.4582 | 40.5713 | 48 | 117.4760 | 40.5605 |
| 21 | 117.4617 | 40.5672 | 49 | 117.4643 | 40.6040 |
| 22 | 117.4679 | 40.5627 | 50 | 117.4578 | 40.6027 |
| 23 | 117.4377 | 40.5675 | 51 | 117.4603 | 40.6073 |
| 24 | 117.4291 | 40.5880 | 52 | 117.4636 | 40.6261 |
| 25 | 117.4268 | 40.5863 | 53 | 117.4668 | 40.6126 |
| 26 | 117.4337 | 40.5904 | 54 | 117.4313 | 40.5823 |
| 27 | 117.4384 | 40.5599 | 55 | 117.5127 | 40.6140 |
| 28 | 117.4459 | 40.5594 | 56 | 117.4739 | 40.5831 |

TableS2 Detail information for transects in Hebei Wulingshan Nature Reserve

| ID | Name | Start | | End | | Distance  (km) | Elevation range  (m) |
| --- | --- | --- | --- | --- | --- | --- | --- |
|  |  | X | Y | X | Y |  |  |
| 1 | Sanchakou | 117.37352 | 40.54062 | 117.41313 | 40.55186 | 6.9 | 630 - 910 |
| 2 | Dongmeisi | 117.49889 | 40.50670 | 117.49483 | 40.54803 | 5.1 | 670 - 910 |
| 3 | Zhuangmugonglu | 117.49769 | 40.64769 | 117.51245 | 40.61887 | 4.1 | 690 - 942 |
| 4 | Beimen | 117.45468 | 40.63492 | 117.46377 | 40.62563 | 3.1 | 677 - 868 |
| 5 | Beigonglu | 117.46372 | 40.62319 | 117.45868 | 40.60797 | 5.7 | 939 - 1386 |
| 6 | Shandingbei | 117.48133 | 40.59798 | 117.46334 | 40.60349 | 12.7 | 1503 - 2118 |
| 7 | Longtanpubu | 117.43872 | 40.60024 | 117.45864 | 40.59611 | 2.8 | 920 - 1490 |
| 8 | Yangtaigou | 117.43357 | 40.60293 | 117.43127 | 40.58233 | 3.1 | 865 - 1223 |
| 9 | Baicaowa | 117.46688 | 40.56446 | 117.47954 | 40.58713 | 7.0 | 1355 - 1759 |
| 10 | Daliushuigou | 117.50969 | 40.62061 | 117.50054 | 40.60027 | 3.8 | 958 - 1394 |
| 11 | Niangniangwa | 117.48691 | 40.56394 | 117.49850 | 40.56952 | 2.5 | 1040 - 1265 |
| 12 | Zhongguyuan | 117.47349 | 40.57102 | 117.48155 | 40.58820 | 4.8 | 1450 - 1753 |
| 13 | Nangonglu | 117.48937 | 40.56033 | 117.47441 | 40.56982 | 7.0 | 1046 - 1465 |
| 14 | Xianrenta | 117.49144 | 40.55145 | 117.46730 | 40.56423 | 2.8 | 942 - 1343 |


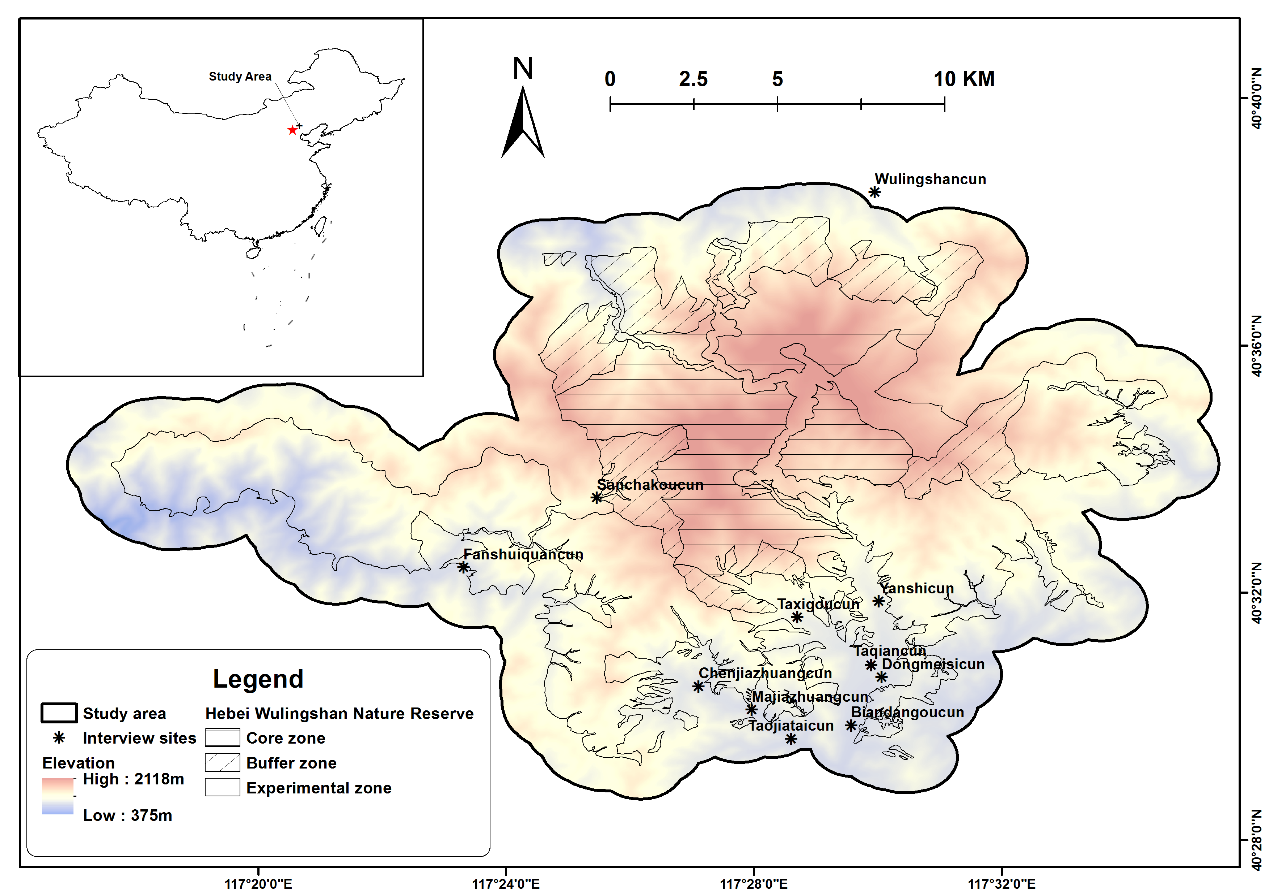


Figure S3 Interview sites in Hebei Wulingshan Nature Reserve (All the villages were around nature reserve).**Climate layers**

Table S3. Eco-geographic variables used in species distribution models in Wulingshan nature reserve for different season.

| ID | Describe | Name | Resource |
| --- | --- | --- | --- |
| 1 | Mean temperature | TEMP | Climate AP |
| 2 | Precipitation | PREC | Climate AP |
| 3 | Precipitation as snow (mm) between August in previous year and July in current year | PAS | Climate AP |
| 4 | Mean Diurnal Range | TRANGE | Climate AP |
| 5 | temperature in summer (Jun, Jul, and Aug) | TMAX_JJA | Climate AP |
| 6 | temperature in summer (Jun, Jul, and Aug) | TMIN_JJA | Climate AP |
| 7 | Precipitation in summer (Jun, Jul, and Aug) | PPT_JJA | Climate AP |
| 8 | temperature in winter (Dec, Jan, and Feb) | TMAX_ DJF | Climate AP |
| 9 | temperature in winter (Dec, Jan, and Feb) | TMIN_ DJF | Climate AP |
| 10 | Precipitation in winter (Dec, Jan, and Feb) | PPT_ DJF | Climate AP |
| 11 | Aspect | ASP | SRTM 90m Digital Elevation Database |
| 12 | Slope | SLO | SRTM 90m Digital Elevation Database |

NOTE: Climate AP v2.03, http://climateap.net/; SRTM 90m Digital Elevation Database, http://srtm.csi.cgiar.org/[1].

For different groups, annual group for resident animals (mammals and resident birds) includes TEMP, PREC, PAS, TRANGE, ASP, and SLO; summer group for summer species (summer birds) includes TMAX_JJA, TMIN_JJA, PPT_JJA, ASP, and SLO; winter group for summer species (summer birds) includes TMAX_JJA, TMIN_JJA, PPT_JJA, ASP, and SLO.

Climate data can be obtained by interpolation of complex multivariate data using thin plate smoothing splines. The fishnet was created in the study area for downloading the climate data by Climate AP[2] (Fig S2). 240 points were obtained for analysis, and the values for mean temperature, precipitation, maximum temperature, and minimum temperature for four seasons per year were downloaded by Climate AP. Due to reduce the bias, we calculate the climate variables from the climate dataset from 1995 to 2015. Environmental variables were calculated with ANUSPLIN ver. 4.36. Mean Diurnal Range were calculated by maximum temperature and minimum temperature. All the variables were obtained by the same cell size (10m*10m).


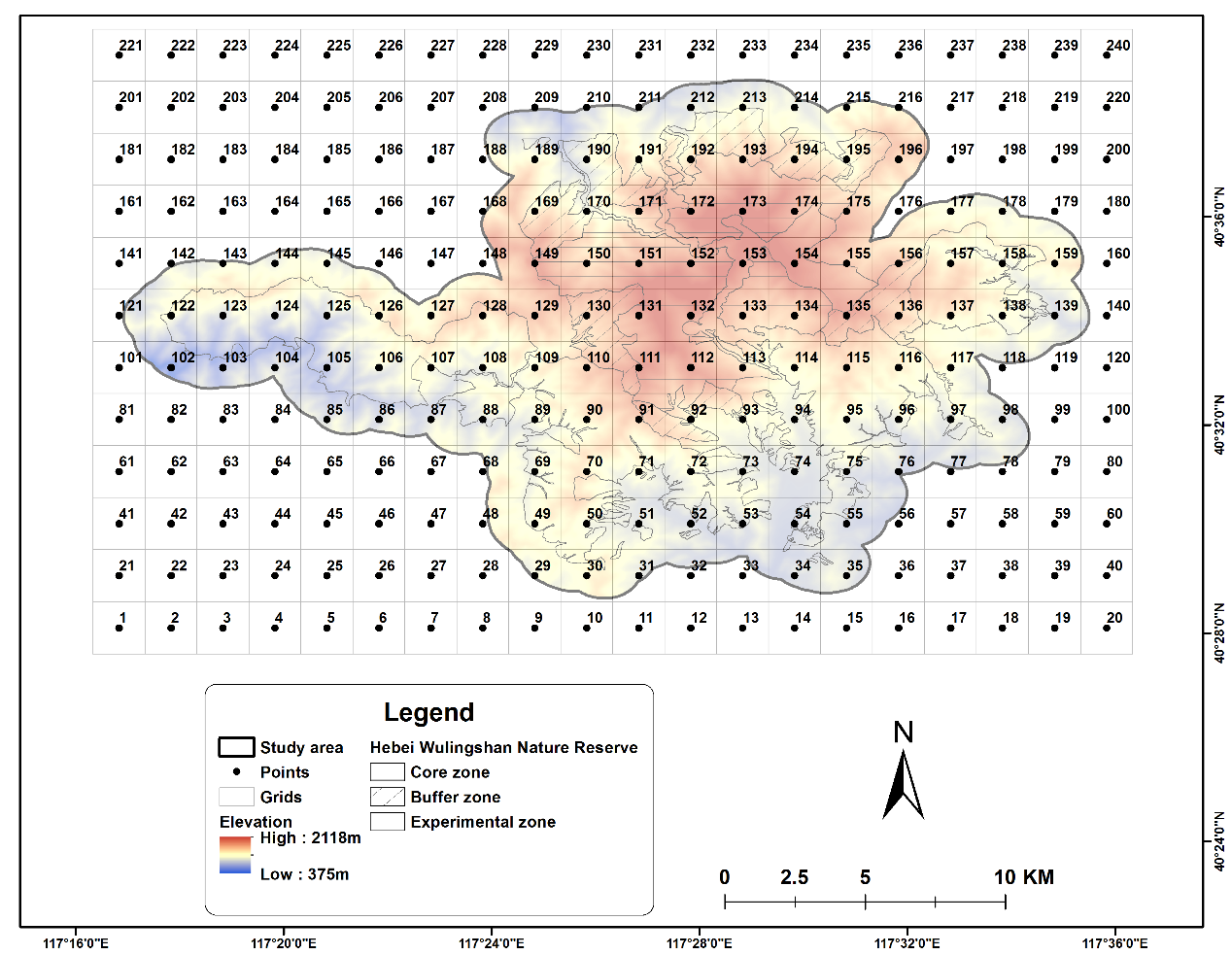


Figure S4 Grids for climate data in Hebei Wulingshan Nature Reserve

**Reference**

[1] Jarvis, A., Reuter, H.I., Nelson, A. & Guevara, E. 2008 *Hole-filled SRTM for the globe Version 4*. USA, CGIAR-CSI SRTM 90m Database.

[2] Wang, T., Hamann, A., Spittlehouse, D.L. & Murdock, T.Q. 2012 ClimateWNA—High-Resolution Spatial Climate Data for Western North America. *Journal of Applied Meteorology and Climatology* 51, 16-29. (doi:10.1175/jamc-d-11-043.1).
