# Supplementary material for: Gap analysis and implications for seasonal management on a local scale
Source: PeerJ. 2018 Sep 20;6:e5622. doi: 10.7717/peerj.5622 (PMC6151258; doi:10.7717/peerj.5622)
Supplement: Supplemental Information 2 [file peerj-06-5622-s002.docx]

**Appendix II Result**

**Species potential distribution**

Table S1 detail result for 64 species in Wulingshan nature reserve

| **ID** | **Species** | **Scientific name** | **Variable importance** | **Range** | **Elevation**  **(m)** | **TSS** | **Group** | **Effective records** | **Conservation** |
| --- | --- | --- | --- | --- | --- | --- | --- | --- | --- |
| 1 | Common Buzzard ^F, I, H^ | *Buteo buteo* | TMAX_DJF | 9.6 | 1418.00 | 0.914 | Winter | 22 | IUCN:LC;CITES:Ⅱ;  RCB:LC |
| 2 | Rough-Legged Buzzard ^F, I, H^ | *Buteo lagopus* | TMIN_DJF | 37.1 | 1350.83 | 0.815 | Winter | 29 | IUCN:LC;CITES:Ⅱ;  RCB:NT |
| 3 | Koklass Pheasant ^C, F, I, H^ | *Pucrasia macrolopha* | MAT | 26.4 | 1488.61 | 0.843 | Annual | 25 | IUCN:LC;RCB:LC |
| 4 | Ring-Necked Pheasant ^C, F, H^ | *Phasianus colchicus* | MAT | 18.8 | 1450.83 | 0.765 | Annual | 25 | IUCN:LC;RCB:LC |
| 5 | Hill Pigeon ^F, I, H^ | *Columba rupestris* | TD | 24.1 | 1151.25 | 0.893 | Annual | 14 | IUCN:LC;RCB:LC |
| 6 | Oriental Turtle Dove ^F, H^ | *Streptopelia orientalis* | MAT | 16.4 | 1023.43 | 0.918 | Annual | 39 | IUCN:LC;RCB:LC |
| 7 | Eurasian Collared Dove ^F, H^ | *Streptopelia decaocto* | PAS | 9.8 | 870.91 | 0.896 | Annual | 18 | IUCN:LC;RCB:LC |
| 8 | Spotted Dove ^F^ | *Streptopelia chinensis* | PAS | 5.5 | 781.01 | 0.919 | Annual | 37 | IUCN:LC;RCB:LC |
| 9 | Oriental Cuckoo ^F, H^ | *Cuculus saturatus* | TMIN_JJA | 19.5 | 1324.17 | 0.916 | Summer | 10 | IUCN:LC;RCB:LC |
| 10 | Fork-Tailed Swift ^F, H^ | *Apus pacificus* | TMIN_JJA | 21.7 | 1551.85 | 0.904 | Summer | 16 | IUCN:LC;RCB:LC |
| 11 | Common Kingfisher ^F, I, H^ | *Alcedo atthis* | MAT | 21.9 | 1149.43 | 0.888 | Annual | 19 | IUCN:LC;RCB:LC |
| 12 | Grey-Headed Woodpecker ^F^ | *Picus canus* | MAT | 29.4 | 882.31 | 0.868 | Annual | 17 | IUCN:LC;RCB:LC |
| 13 | Great Pied Woodpecker ^F^ | *Dendrocopos major* | MAT | 46.2 | 1353.69 | 0.764 | Annual | 16 | IUCN:LC;RCB:LC |
| 14 | White-Backed Woodpecker ^F, H^ | *Dendrocopos leucotos* | MAT | 25.1 | 1156.91 | 0.904 | Summer | 51 | IUCN:LC;RCB:LC |
| 15 | Pigmy Woodpecker ^F^ | *Dendrocopos kizuki* | MAT | 40.9 | 1069.84 | 0.797 | Annual | 34 | IUCN:LC;RCB:LC |
| 16 | Barn Swallow ^F, H^ | *Hirundo rustica* | PPT_JJA | 23.3 | 881.42 | 0.902 | Summer | 18 | IUCN:LC;RCB:LC |
| 17 | Red-Rumped Swallow ^F, H^ | *Cecropis daurica* | PPT_JJA | 22.3 | 871.49 | 0.894 | Summer | 15 | IUCN:LC;RCB:LC |
| 18 | Asian House Martin ^F^ | *Delichon dasypus* | TMIN_JJA | 14.3 | 1595.87 | 0.929 | Summer | 33 | IUCN:LC;RCB:LC |
| 19 | Grey Wagtail ^F, H^ | *Motacilla cinerea* | TMIN_JJA | 24.3 | 1036.82 | 0.898 | Summer | 33 | IUCN:LC;RCB:LC |
| 20 | White Wagtail ^F, I, H^ | *Motacilla alba* | PPT_JJA | 44.8 | 980.50 | 0.788 | Summer | 22 | IUCN:LC;RCB:LC |
| 21 | Chinese Bulbul ^F^ | *Pycnonotus sinensis* | MAT | 16.5 | 1342.40 | 0.929 | Annual | 35 | IUCN:LC;RCB:LC |
| 22 | Eurasian Jay ^C, F, I^ | *Garrulus glandarius* | MAT | 15.0 | 996.02 | 0.859 | Annual | 17 | IUCN:LC;RCB:LC |
| 23 | Red-Billed Blue Magpie ^F, H^ | *Urocissa erythrorhyncha* | MAT | 14.1 | 881.31 | 0.9 | Annual | 34 | IUCN:LC;RCB:LC |
| 24 | Common Magpie ^F, I, H^ | *Pica pica* | PAS | 25.1 | 828.05 | 0.888 | Annual | 35 | IUCN:LC;RCB:LC |
| 25 | Spotted Nutcracker ^F^ | *Nucifraga caryocatactes* | MAT | 26.5 | 1288.53 | 0.818 | Annual | 35 | IUCN:LC;RCB:LC |
| 26 | Large-Billed Crow ^F, H^ | *Corvus macrorhynchos* | MAT | 30.4 | 1181.08 | 0.782 | Annual | 43 | IUCN:LC;RCB:LC |
| 27 | Brown Dipper ^F^ | *Cinclus pallasii* | MAT | 16.9 | 1348.05 | 0.923 | Annual | 18 | IUCN:LC;RCB:LC |
| 28 | Winter Wren ^F^ | *Troglodytes troglodytes* | MAT | 23.3 | 1324.26 | 0.868 | Annual | 50 | IUCN:LC;RCB:LC |
| 29 | Alpine Accentor ^F, I^ | *Prunella collaris* | TMIN_JJA | 15.8 | 1607.99 | 0.919 | Summer | 11 | IUCN:LC;RCB:LC |
| 30 | Mountain Accentor ^F, I^ | *Prunella montanella* | TMIN_DJF | 5.2 | 1360.08 | 0.895 | Winter | 13 | IUCN:LC;RCB:LC |
| 31 | Daurian Redstart ^F^ | *Phoenicurus auroreus* | TMIN_JJA | 43.6 | 1064.96 | 0.779 | Summer | 66 | IUCN:LC;RCB:LC |
| 32 | Plumbeous Water Redstart ^F^ | *Rhyacornis fuliginosa* | MAT | 20.2 | 1177.97 | 0.906 | Annual | 32 | IUCN:LC;RCB:LC |
| 33 | Blue Whistling Thrush ^F^ | *Myophonus caeruleus* | TMIN_JJA | 15.6 | 1395.93 | 0.913 | Summer | 15 | IUCN:LC;RCB:LC |
| 34 | Grey-Sided Thrush ^F^ | *Turdus feae* | TMIN_JJA | 19.1 | 1123.78 | 0.916 | Summer | 16 | IUCN:VU;RCB:VU |
| 35 | Dusky Thrush ^F, I, H^ | *Turdus eunomus* | TMIN_DJF | 35.0 | 1104.65 | 0.844 | Winter | 11 | RCB:LC |
| 36 | Plain Laughingthrush ^F^ | *Garrulax davidi* | MAT | 52.2 | 1128.80 | 0.754 | Annual | 37 | IUCN:LC;RCB:LC |
| 37 | Vinous-Throated Parrotbill ^F^ | *Paradoxornis webbianus* | MAT | 15.7 | 870.68 | 0.926 | Annual | 26 | IUCN:LC;RCB:LC |
| 38 | Asian Stubtail ^F^ | *Urosphena squameiceps* | TMIN_JJA | 10.5 | 981.26 | 0.887 | Summer | 50 | IUCN:LC;RCB:LC |
| 39 | Yellow-Browed Warbler ^F^ | *Phylloscopus inornatus* | TMIN_JJA | 28.2 | 1058.67 | 0.793 | Summer | 30 | IUCN:LC;RCB:LC |
| 40 | Pallas's Leaf Warbler ^F^ | *Phylloscopus proregulus* | TMIN_JJA | 26.5 | 1005.34 | 0.876 | Summer | 24 | IUCN:LC;RCB:LC |
| 41 | Arctic Warbler ^F^ | *Phylloscopus borealis* | TMIN_JJA | 21.3 | 1197.72 | 0.854 | Summer | 41 | IUCN:LC;RCB:LC |
| 42 | Large-Billed Leaf Warbler ^F^ | *Phylloscopus magnirostris* | TMIN_JJA | 10.0 | 1472.14 | 0.908 | Summer | 15 | IUCN:LC;RCB:LC |
| 43 | Blyth's Leaf Warbler ^F^ | *Phylloscopus reguloides* | TMIN_JJA | 16.4 | 1174.52 | 0.783 | Summer | 37 | IUCN:LC;RCB:LC |
| 44 | Yellow-Bellied Tit ^F^ | *Parus venustulus* | TMIN_JJA | 31.9 | 1206.97 | 0.805 | Summer | 114 | IUCN:LC;RCB:LC |
| 45 | Coal Tit ^F^ | *Parus ater* | MAT | 20.5 | 1496.59 | 0.902 | Annual | 15 | IUCN:LC;RCB:LC |
| 46 | Marsh Tit ^F^ | *Parus palustris* | MAT | 17.3 | 915.20 | 0.918 | Annual | 43 | IUCN:LC;RCB:LC |
| 47 | Willow Tit ^F^ | *Parus songarus* | MAT | 25.3 | 1316.18 | 0.873 | Annual | 118 | IUCN:LC;RCB:LC |
| 48 | Long-Tailed Tit ^F^ | *Aegithalos caudatus* | TMIN_DJF | 17.8 | 1327.13 | 0.849 | Winter | 59 | RCB:LC |
| 49 | Eurasian Nuthatch ^F^ | *Sitta europaea* | MAT | 26.0 | 1396.36 | 0.886 | Annual | 27 | IUCN:LC;RCB:LC |
| 50 | Eurasian Tree Sparrow ^F, H^ | *Passer montanus* | PAS | 4.7 | 781.12 | 0.84 | Annual | 23 | IUCN:LC;RCB:LC |
| 51 | Grey-Capped Greenfinch ^F, I^ | *Carduelis sinica* | MAT | 26.4 | 1033.91 | 0.884 | Annual | 28 | IUCN:LC;RCB:LC |
| 52 | Leopard Cat ^C, F, I, H^ | *Prionailurus bengalensis* | MAT | 21.8 | 1441.83 | 0.866 | Annual | 21 | IUCN:LC;CITES:Ⅱ;  RCB:VU |
| 53 | Hog Badger ^C, F, I, H^ | *Arctonyx collaris* | MAT | 30.6 | 1467.81 | 0.866 | Annual | 39 | IUCN:LC;RCB:NT |
| 54 | Siberian Roe Deer ^C, F, I, H^ | *Capreolus pygargus* | MAT | 34.7 | 1498.36 | 0.838 | Annual | 36 | IUCN:LC;RCB:NT |
| 55 | Raccoon-Dog ^C, F, I, H^ | *Nyctereutes procyonoides* | MAT | 27.2 | 1475.20 | 0.882 | Annual | 15 | IUCN:LC;RCB:NT |
| 56 | Chinese Goral ^C, F, I, H^ | *Naemorhedus griseus* | MAT | 30.0 | 1483.15 | 0.864 | Annual | 27 | IUCN:VU;RCB:VU |
| 57 | Eurasian Red Squirrel ^C, F, I, H^ | *Sciurus vulgaris* | MAT | 35.1 | 1492.99 | 0.846 | Annual | 15 | IUCN:LC;RCB:NT |
| 58 | Pere David's Rock Squirrel ^C, F, I, H^ | *Sciurotamias davidianus* | MAT | 31.9 | 1440.09 | 0.787 | Annual | 11 | IUCN:LC;RCB:LC |
| 59 | Wild Boar ^C, F, I, H^ | *Sus scrofa* | MAT | 16.3 | 1389.08 | 0.902 | Annual | 29 | IUCN:LC;RCB:LC |
| 60 | Tolai Hare ^C, F, I, H^ | *Lepus tolai* | MAT | 34.1 | 1428.39 | 0.836 | Annual | 19 | IUCN:LC;RCB:LC |
| 61 | Siberian Chipmunk ^C, F, I, H^ | *Tamias sibiricus* | MAT | 20.1 | 1470.19 | 0.92 | Annual | 13 | IUCN:LC;RCB:LC |
| 62 | Siberian Weasel ^C, F, I, H^ | *Mustela sibirica* | MAT | 32.5 | 1491.30 | 0.844 | Annual | 13 | IUCN:LC;RCB:LC |
| 63 | Amur Hedgehog ^C, F, I, H^ | *Erinaceus amurensis* | MAT | 28.4 | 1453.29 | 0.864 | Annual | 18 | IUCN:LC;RCB:LC |
| 64 | Asian Badger ^C, F, I, H^ | *Meles leucurus* | MAT | 43.8 | 1456.07 | 0.74 | Annual | 16 | IUCN:LC;RCB:NT |

Note: Record resources: “^C^” means camera records; “^F^” mean filed survey (bird survey); “^I^” means interview; “^H^” means specimen records. Variable importance mean the variable important value is highest among variables (obtain from BIOMOD2). TSS were obtained from BIOMOD2. Range means potential distribution for each species. Elevation means mean elevation for potential distribution. Effective records mean occurrence points were used for modelling. IUCN Red List categories follow IUCN (2017); CITES categories obtained from Species+ (https://www.speciesplus.net/); Redlist categories obtained from Redlist of China’s Biodiversity (<http://www.zhb.gov.cn/gkml/hbb/bgg/201505/t20150525_302233.htm>).

Table S2 Information for species which excluded for this study

| **ID** | **Species** | **Scientific name** | **Exclude reason** | **ID** | **Species** | **Scientific name** | **Exclude reason** |
| --- | --- | --- | --- | --- | --- | --- | --- |
| 1 | Large Hawk-Cuckoo | *Hierococcyx sparverioides* | A | 20 | Japanese White-eye | *Zosterops japonicus* | A |
| 2 | Indian Cuckoo | *Cuculus micropterus* | A;B | 21 | Manchurian Bush Warbler | *Horornis canturians* | A;B |
| 3 | Oriental Honey Buzzard | *Pernis ptilorhynchus* | B | 22 | Eyebrowed Thrush | *Turdus obscurus* | A;B |
| 4 | Eurasian Sparrowhawk | *Accipiter nisus* | B | 23 | Orange-flanked Bush-Robin | *Tarsiger cyanurus* | A;B |
| 5 | Pied Harrier | *Circus melanoleucos* | B | 24 | White-bellied Redstart | *Luscinia phoenicuroides* | A |
| 6 | Grey-faced Buzzard Eagle | *Butastur indicus* | A;B | 25 | Green-backed Flycatcher | *Ficedula elisae* | A;B |
| 7 | Scops Owl | *Otus scops* | A;B | 26 | Blue-and-white Flycatcher | *Cyanoptila cyanomelana* | A |
| 8 | Red-footed Falcon | *Falco vespertinus* | B | 27 | Pechora Pipit | *Anthus gustavi* | A |
| 9 | Eurasian Hobby | *Falco subbuteo* | B | 28 | Orienfnl Tree Pipit | *Anthus hodgsoni* | A |
| 10 | Peregrine Falcon | *Falco peregrinus* | A | 29 | Brambling | *Fringilla montifringilla* | B |
| 11 | Long-tailed Minivet | *Pericrocotus ethologus* | B | 30 | Godlewski's Bunting | *Emberiza godlewskii* | A |
| 12 | Carrion Crow | *Corvus corone* | A | 31 | Little Bunting | *Emberiza pusilla* | B |
| 13 | Japanese Tit | *Parus minor* | A | 32 | Yellow-throated Bunting | *Emberiza elegans* | B |
| 14 | Common House Martin | *Delichon urbicum* | A;B | 33 | Black-faced Bunting | *Emberiza spodocephala* | A;B |
| 15 | Dusky Warbler | *Phylloscopus fuscatus* | A | 34 | Red fox | *Vulpes vulpes* | C |
| 16 | Yellow-streaked Warbler | *Phylloscopus armandii* | A | 35 | Yellow-throated marten | *Martes flavigula* | C |
| 17 | Chinese Leaf Warbler | *Phylloscopus yunnanensis* | A;B | 36 | Masked palm civet | *Paguma larvata* | C |
| 18 | Hume's Leaf Warbler | *Phylloscopus humei* | A | 37 | Leopard | *Panthera pardus* | C |
| 19 | Eastern Crowned Warbler | *Phylloscopus coronatus* | B |  |  |  |  |

Note: A. Effective occurrence points were shortage and not enough for environmental niche modeling (less than 10).

B. Passing birds and straggler birds were excluded for this research.

C. Only have historical data without any effective record in this research.

**Elevation**

**
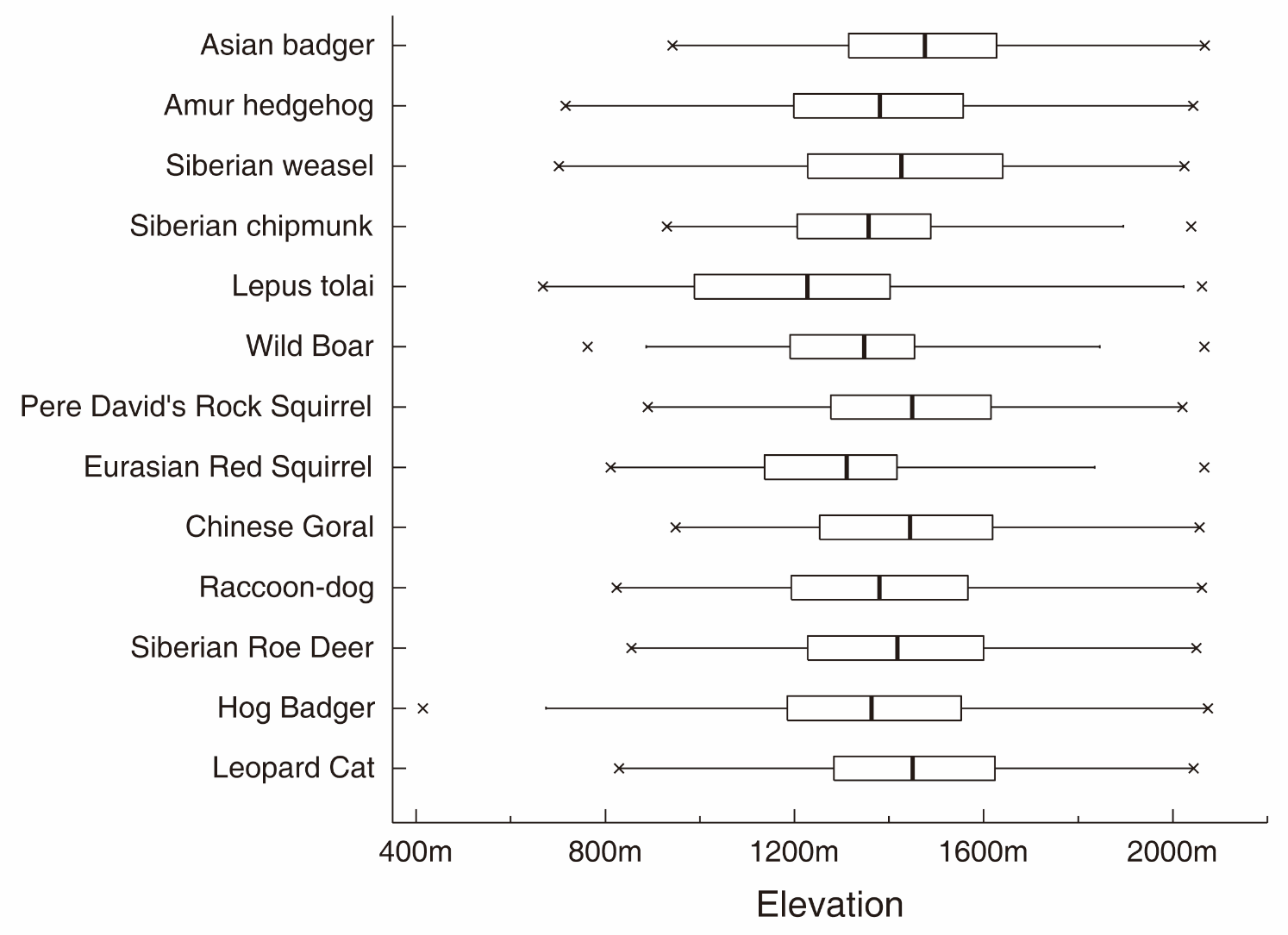
**

Figure S1 Elevation range of mammals in Hebei Wulingshan Nature Reserve.

Note: Black line means mean elevation; “×” mean extremum


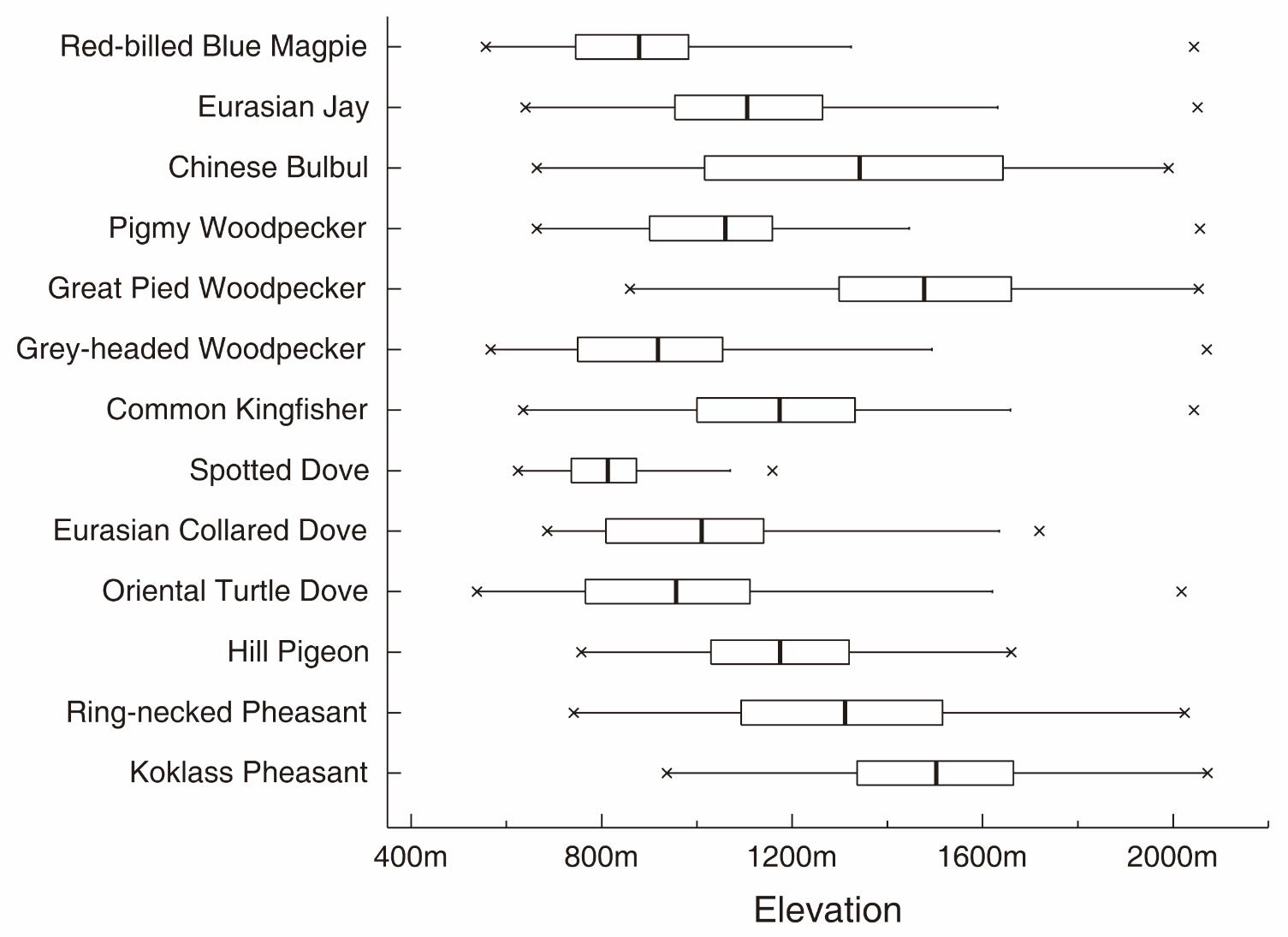


Figure S2 Elevation range of resident birds in Hebei Wulingshan Nature Reserve (part1)

Note: Black line means mean elevation; “×” mean extremum


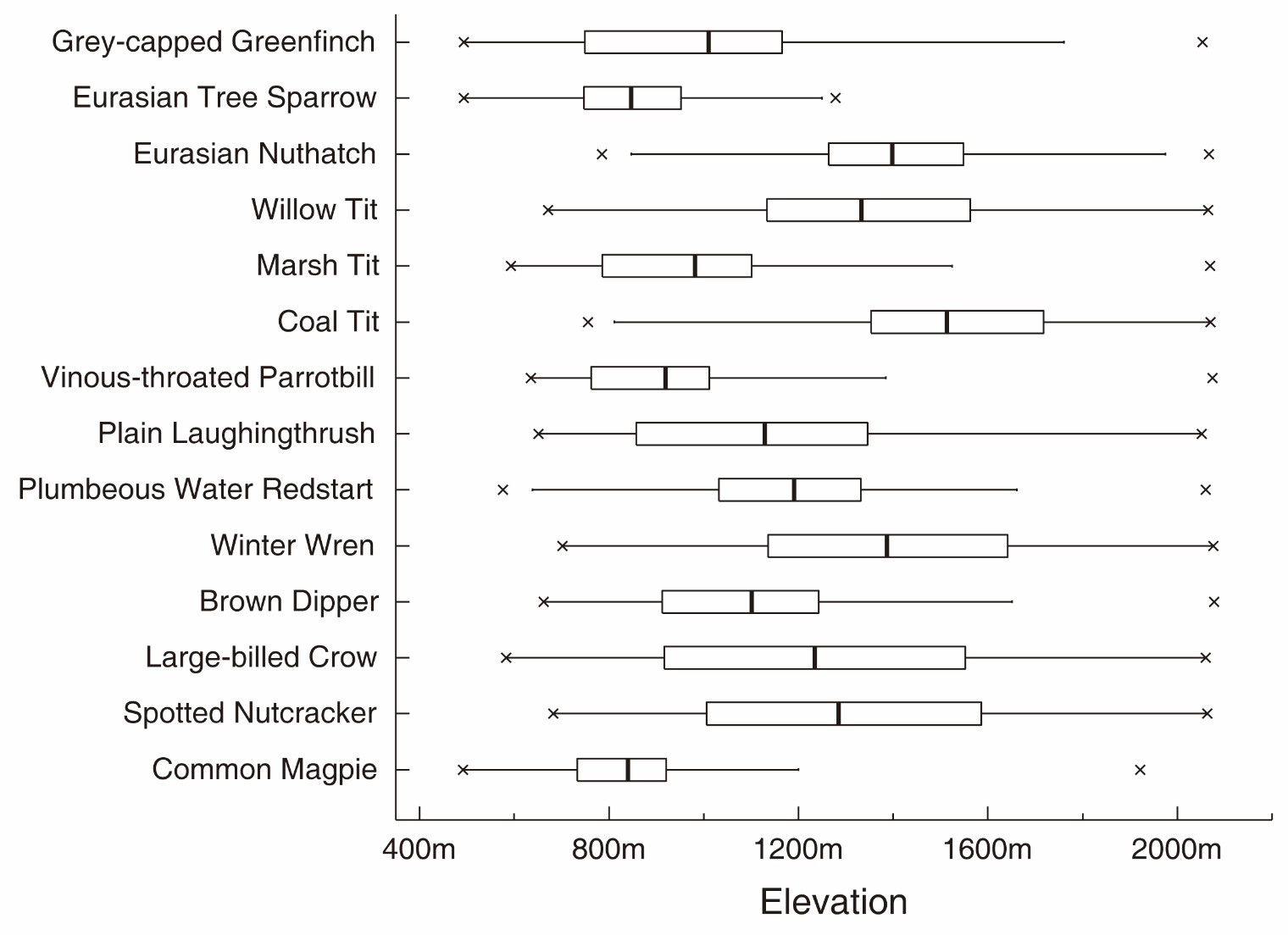


Figure S2 Elevation range of resident birds in Hebei Wulingshan Nature Reserve (part2)

Note: Black line means mean elevation; “×” mean extremum

**
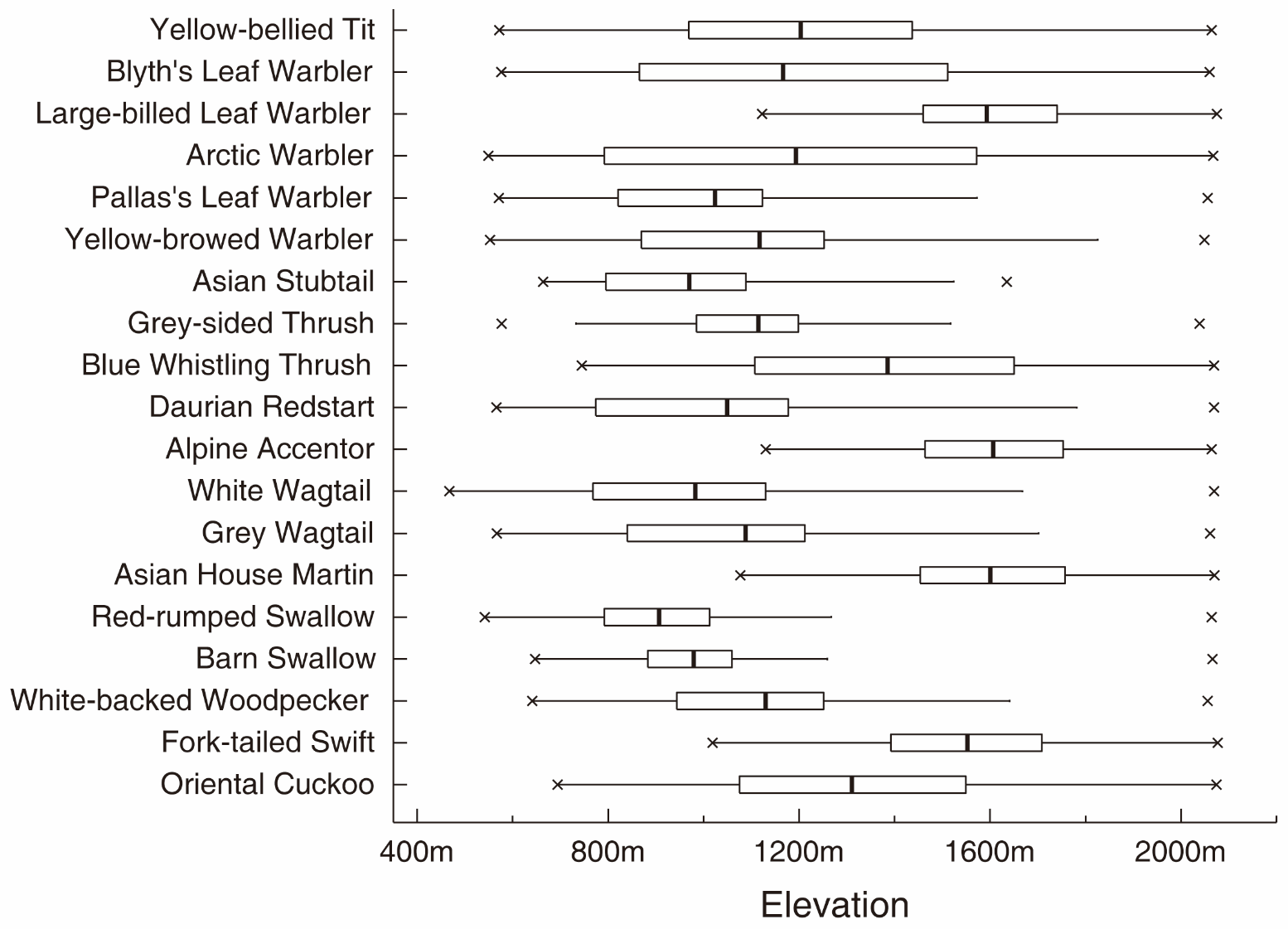
**

Figure S3 Elevation range of summer birds in Hebei Wulingshan Nature Reserve

Note: Black line means mean elevation; “×” mean extremum

**
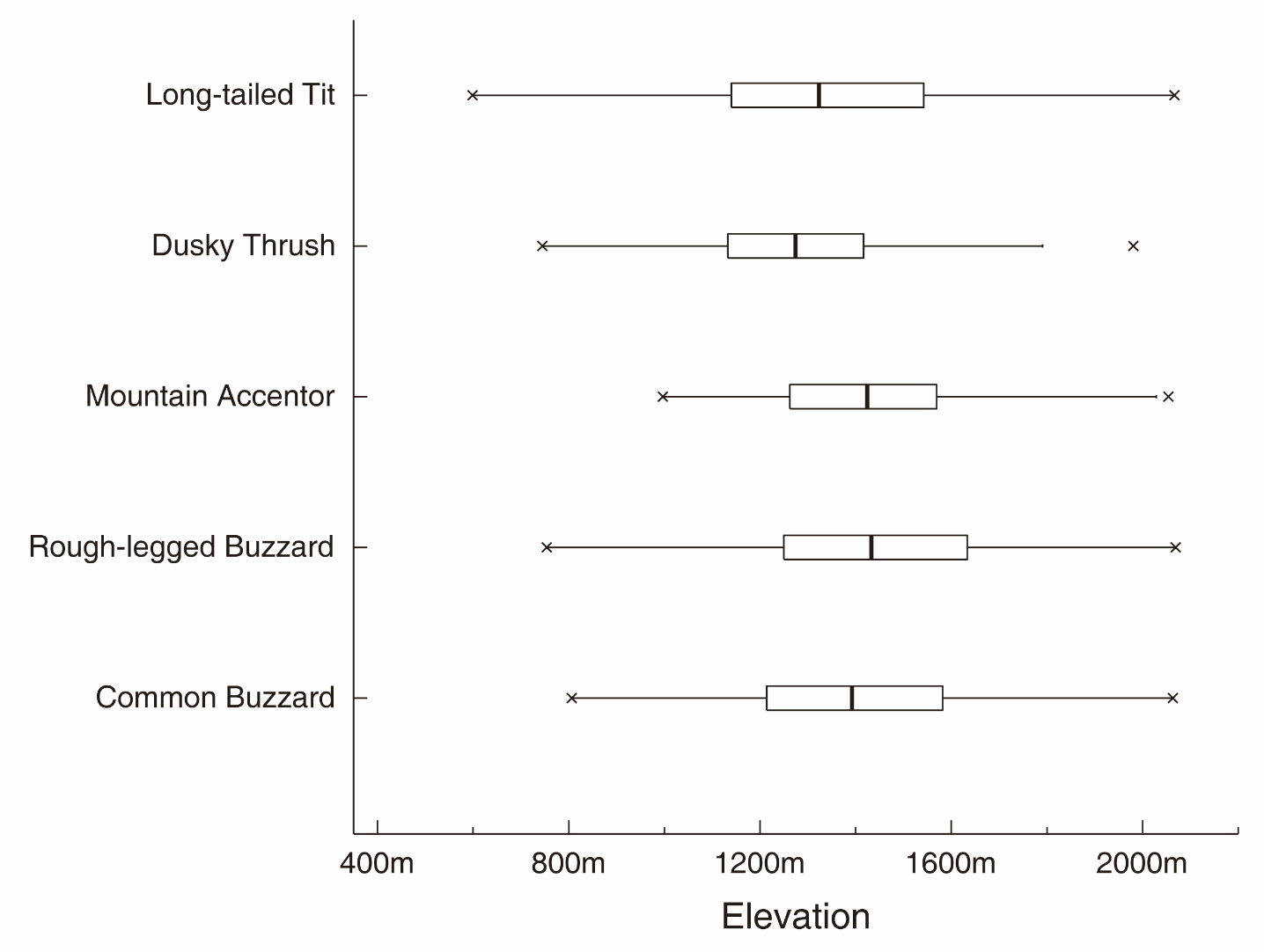
**

Figure S4 Elevation range of winter birds in Hebei Wulingshan Nature Reserve

Note: Black line means mean elevation; “×” mean extremum

**Conservation**

Table S2 Relationship between nature reserve and hotspot

| Area | Conservation | All Species | | Resident | | Summer | | Winter | |
| --- | --- | --- | --- | --- | --- | --- | --- | --- | --- |
|  |  | Area | Persentage | Area | Persentage | Area | Persentage | Area | Persentage |
| Core Zone | Priority | 0.994 | 69.40% | 1.229 | 67.41% | 0.933 | 71.50% | 1.086 | 67.33% |
| Buffer Zone | Priority | 0.004 | 0.31% | 0.031 | 1.67% | 0.000 | 0.02% | 0.029 | 1.78% |
| Experimental Zone | Priority | 0.398 | 27.78% | 0.493 | 27.04% | 0.346 | 26.51% | 0.434 | 26.92% |
| 1km around Nature Reserve | Priority | 0.036 | 2.52% | 0.071 | 3.88% | 0.026 | 1.97% | 0.064 | 3.97% |
| Core Zone | Important | 11.049 | 51.52% | 5.671 | 62.41% | 5.038 | 65.16% | 11.718 | 53.38% |
| Buffer Zone | Important | 2.130 | 9.93% | 0.716 | 7.88% | 0.436 | 5.64% | 2.455 | 11.18% |
| Experimental Zone | Important | 5.301 | 24.72% | 2.007 | 22.09% | 1.617 | 20.92% | 5.464 | 24.89% |
| 1km around Nature Reserve | Important | 2.964 | 13.82% | 0.693 | 7.62% | 0.640 | 8.28% | 2.314 | 10.54% |

Table S3 Suggestion for conservation action

| **ID** | **Period** | **Location** | **Action** | | | |
| --- | --- | --- | --- | --- | --- | --- |
|  |  |  | **Monitor** | **Enforcement** | **Popular science** | **Cooperate with local people** |
| 1 | Annual | Around | ⚫ |  |  |  |
| 2 | Annual | Around |  |  | ⚫ | ⚫ |
| 3 | Annual | Around |  |  | ⚫ |  |
| 4 | Summer; Winter | Around |  |  | ⚫ | ⚫ |
| 5 | Summer; Winter | Around |  |  | ⚫ |  |
| 6 | Summer | Around | ⚫ |  | ⚫ | ⚫ |
| 7 | Annual | In |  | ⚫ | ⚫ |  |
| 8 | Annual | In |  | ⚫ |  |  |
| 9 | Annual | In | ⚫ | ⚫ | ⚫ |  |
| 10 | Annual | In | ⚫ | ⚫ | ⚫ |  |
| 11 | Annual | In | ⚫ | ⚫ | ⚫ |  |
| 12 | Annual | In | ⚫ | ⚫ | ⚫ |  |

Note: Due to deep snow and strict management in winter in Hebei Wulingshan Nature Reserve, potential area for conservation action tend to consider area around nature reserve in winter. For location, “In” means area located in nature reserve, and “Around” means area located outside or around the nature reserve.

**Photos**

**
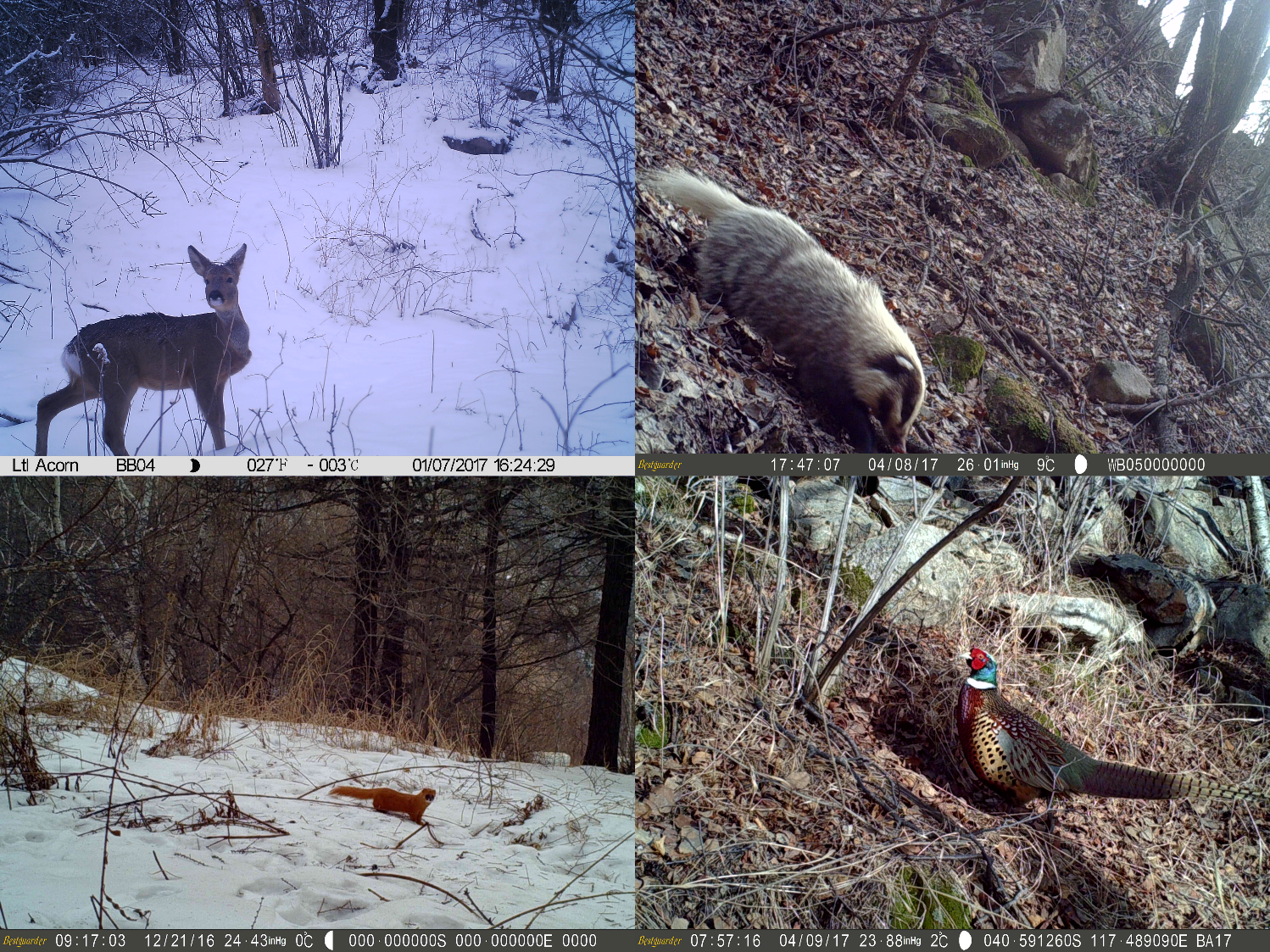
**

Figure S5 Photography from camera trapping

**
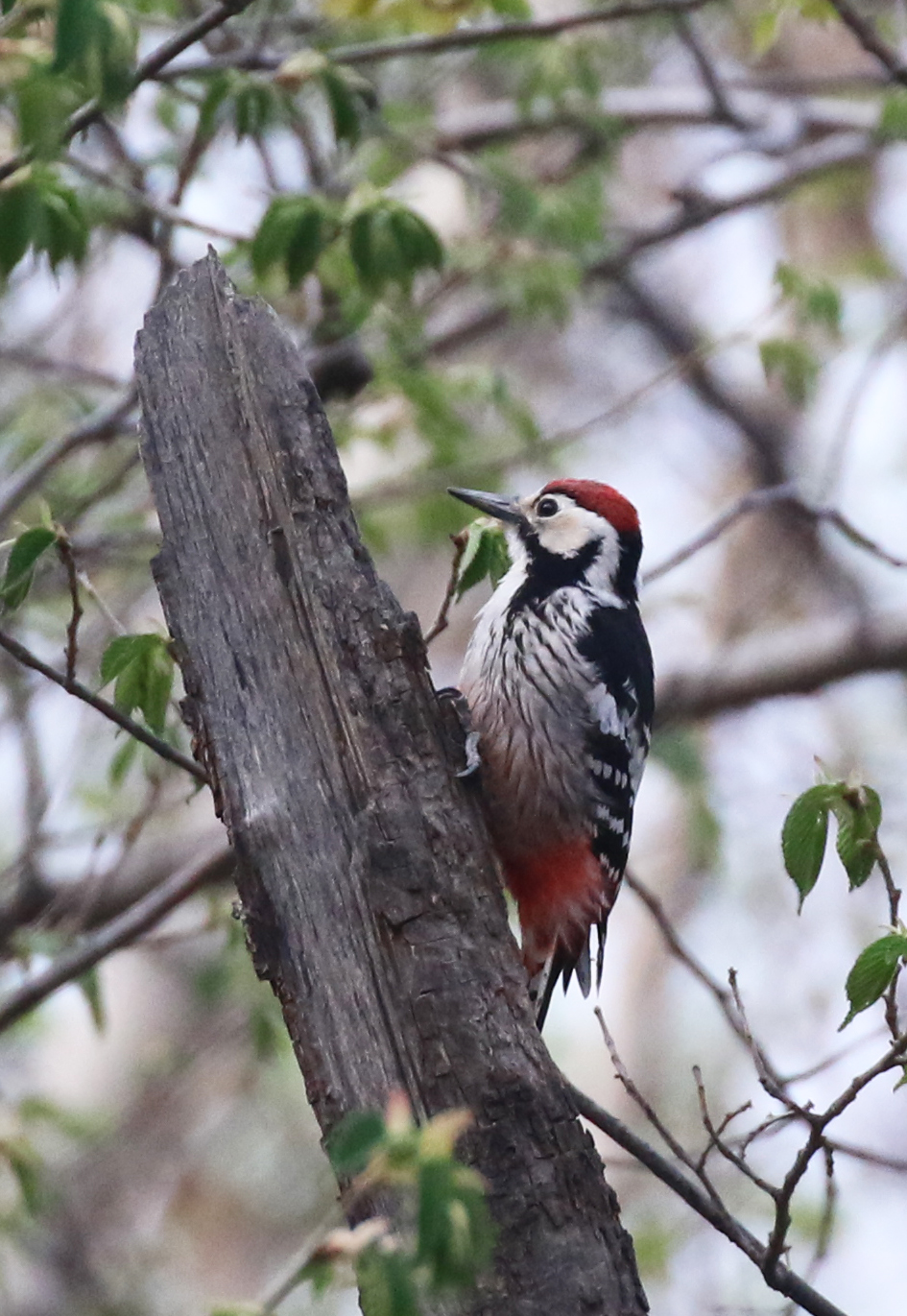
**

Figure S6 Photography from bird survey (*Dendrocopos leucotos*)
